# Comparison of the Effect of Bioglass, Chitosan, and SDF Compounds on Remineralization of Primary Caries Lesions in Primary Teeth: An *in vitro* Study

**DOI:** 10.30476/dentjods.2023.97954.2041

**Published:** 2024-09-01

**Authors:** Razieh Meshki, Nazgol Ghahramani, Maryam Kouchak, Shirin Taravati

**Affiliations:** 1 Dept. of Pediatric Dentistry, School of Dentistry Ahvaz Jundishapur University of Medical Sciences, Ahvaz, Iran; 2 Dept. of Pediatric Dentistry, Ahvaz Jundishapur University of Medical Sciences, Ahvaz, Iran; 3 Dept. of Pharmaceutics, Nanotechnology Research Center, Ahvaz Jundishapur University of Medical Sciences, Ahvaz, Iran

**Keywords:** Deciduous, Dental Enamel, Tooth, Tooth Remineralization

## Abstract

**Statement of the Problem:**

Dental caries are among the most common oral and dental diseases affecting adults and children. To prevent caries, either the factors that cause caries should be reduced or the host resistance should be increased. Several compounds, such as bioglass, chitosan, and silver diamine fluoride (SDF), can enhance enamel remineralization.

**Purpose:**

This study was conducted to investigate the effects of chitosan, bioglass, chitosan-bioglass, and SDF compounds on remineralizing primary enamel lesions.

**Materials and Method:**

In this *in vitro* study, seventy-two primary canine teeth were collected. The teeth were exposed to a demineralization solution for 72 hours to create primary caries lesions. The primary Vickers microhardness test (VMT) was conducted to measure the initial values. The samples were randomly divided into six groups (n=12): Group 1: bioglass-chitosan solution; Group 2: chitosan; Group 3: bioglass solution; Group 4: SDF; Group 5: remineralization solution; Group 6: distilled water. The solutions of Groups 1, 2, and 3 were applied to the samples for 7 days, while the SDF solution was applied only once. The samples were immersed in an artificial saliva solution, which was refreshed daily. After the treatment, the final Vickers microhardness test (VMT) values were recorded. The data were analyzed statistically using a two-way ANOVA and Tukey's test (*p*< 0.05).

**Results:**

The results indicated a statistically significant effect of remineralizing compounds on both pre-treatment and post-treatment microhardness (*p*< 0.0001). However, no significant difference in microhardness was observed between the groups studied (*p*= 0.225).

**Conclusion:**

All the compounds utilized in this study demonstrated a significant remineralizing effect on enamel lesions caused by primary caries in primary teeth. The chitosan-bioglass and bioglass groups exhibited the highest levels of remineralization, respectively. However, the comparison between the groups yielded insignificant results due to the dispersion of the samples. Therefore, further studies with larger sample sizes are recommended.

## Introduction

Thirty to sixty percent of preschool children worldwide experience early childhood caries, which is a chronic infectious disease [ 1 ]. It spreads rapidly and can lead to intense pain, facial esthetic concerns, swelling [ 2 ], and impaired speech in children up to 71 months of age [ 1 , 3 ]. Caries occur when there is an imbalance between re-mineralization and demineralization, leading to changes in the chemical structure and morphology of tooth enamel [ 4 ]. The conventional restorative approach for treating primary caries poses significant challenges, particularly in uncooperative patients. Therefore, a non-invasive treatment option that can halt the progression of demineralization in the early stages of caries may be a preferable choice [ 5 ].

Recently, several investigations have used remineralizing materials in dentistry [ 6 - 7 ]. Chitosan is one such compound that has undergone thorough examination regarding its diverse properties [ 7 ].

Chitosan and its derivatives have been reported as a novel class of new biomaterials, because of their excelent biocompatibility, multifunctional biological effect, remineralizing, and antibacterial impacts on tooth structure [ 8 ].

Bioglass is another recently introduced compound for lesion remineralization [ 9 ]. When exposed to saliva or other physiological fluids, bioglass triggers apatite formation on the outer surface of tooth enamel [ 10 ]. Hydroxyapatite is the primary apatite formed from bioglass. Additionally, fluorapatite can be generated when bioglasses incorporate fluoride [ 11 ].

According to laboratory-based results, bioactive glasses are comparable with topical fluoride and milk protein-derived casein phosphopeptide-amorphous calcium phosphate (CPP-ACP) regarding their remineralization effects [ 12 ]. Therefore, bioactive glasses increase enamel remineralization faster and more effectively. Nonetheless, there is a need for clinical trials to confirm their effects [ 13 ].

Some other remineralization and antimicrobial compounds contain silver (Ag), like silver diamine fluoride (SDF), with a bactericidal effect. SDF is also safe, affordable and easy to use to stop the progression of caries [ 14 ]. According to clinical trials, a topically applied SDF solution suppresses demineralization [ 15 ].

SDF has a pH of 10. It is postulated that SDF reacts with hydroxyapatite at its alkaline pH, resulting in the formation of fluorapatite. Fluorapatite, being less soluble than hydroxyapatite in acidic environments, is believed to contribute to the caries prevention properties of SDF [ 16 ]. Literature suggests that SDF is more effective in preventing caries compared to fluoride varnish. Based on compelling evidence, SDF has demonstrated the ability to halt caries lesions in approximately 80% of cases [ 17 ].

Given the significance of caries prevention in pediatric dentistry, this study aims to explore the impact of chitosan, bioglass, chitosan-bioglass, and SDF on the remineralization of artificial enamel caries. The objective is to identify novel and effective compounds for remineralizing primary enamel lesions in primary teeth.

## Materials and Method

### Sampling

The ethics code for conducting this study was obtained from the Ethics Committee of Jundishapur University of Medical Sciences (IR.AJUMS.REC.1400.697).

In this *in vitro* study, dental samples were collected over two months from the Dentistry Department of Jundishapur University of Medical Sciences in Ahvaz and private clinics. The samples were then immersed in a 0.9% sodium chloride solution at room temperature. According to Zhang *et al*. [ 5 ], the sample size was 72 teeth with a test power of 80%. The primary canine teeth selected for the study were all intact, without caries, fractures, cracks, hypoplasia, or restorations, and were extracted for orthodontic purposes. A total of six groups were included in the study, with 12 samples allocated to each group. Prior to commencing the experiment, the samples were disinfected by immersing them in a 0.1% thymol solution (Aldrich, USA) for 48 hours. The surface of the samples was cleaned using sterile gas and brushed with a low-speed handpiece. Subsequently, the specimens were kept in a 0.9% sodium chloride solution at room temperature until the initiation of the investigation [ 18 ]. Using a high-speed handpiece equipped with a diamond fissure bur (Tizkavan, Iran), samples were cut from the cementoenamel junction (CEJ) area. Subsequently, the tooth crowns were fixed to epoxy resin cubes (Acropars 2000, Iran; dimensions: 1cm x 1cm x 1.5cm). This positioning ensured that only a 2x2 mm window of the labial surface of the teeth remained exposed and parallel to the surface of the resin [ 19 ]. Random numbers ranging from 1 to 72 were marked on the lower portion of each cube. 

### Forming Artificial Caries

The demineralization solution, which was used to induce primary caries lesions in the samples, was prepared at the laboratory of the Department of Pharmacy, Jundishapur University, Ahvaz. The demineralizing solution consisted of 8.8 mM KH_2_PO_4_, 0.2 M acetic acid, and 8.8 mM CaCl_2_, with a pH of 4.4 that was adjusted using 4M KOH [ 20 ]. The samples were placed in the demineralization solution for 72 h [ 21 ]. An incubator (Innova-USA) maintained the temperature of the solution at 37°C. A pH meter (Metrohm, Swiss) was employed to monitor the pH of the solution on a daily basis [ 21 ]. After three days, the specimens were removed from the solution, followed by rinsing with distilled water and air-drying gently.

### Vickers microhardness Test

The Vickers hardness test machine (Innovatest, Netherlands) was utilized to collect data in this study. The primary and secondary microhardness tests were conducted at three points on the surface of each specimen that was 100 microns apart, applying a 50 g force for 10 seconds [ 19 ]. The average of these measurements was reported as the Vickers hardness number (VHN). The primary microhardness was measured before the application of remineralization compounds, while the secondary microhardness was measured after their application.

### Assigning Groups

The samples were randomly assigned to six groups (n=12) after recording the primary microhardness. Each group was treated with the following compounds:

Group 1 (Bioglass-Chitosan group): A mixture of 1g bioglass nanoparticle 45S5 (synthesized according to previous studies) [ 22 - 23 ] and 2ml chitosan (Primex, Iceland)

Group 2 (Chitosan group): Chitosan solution 2.5% (25mg in 10ml of 0.1 M acetic acid)

Group 3 (Bioglass group): Bioglass solution 6% (Mixed 0.6g 45S5 BAG nanoparticles with 9.4g of distilled water)

Group 4 (SDF group): SDF 30% (Biodinamica, Brazil CARIESTOP)

Group 5(Positive control): Remineralization solution 

Group 6 (Negative control): Distilled water 

### Remineralization Regime

The solutions of groups 1, 2, and 3 were applied daily for 3 minutes over seven days [ 24 ] at room temperature using a micro brush (Premium Plus, China). The SDF solution was applied only once for 3 minutes [ 17 ]. Throughout this period, the samples were immersed in a fresh artificial saliva solution that was renewed daily.

The composition of the artificial saliva solution included 10 g/l Sodium Carboxymethyl Cellulose, 2g/l Methyl-phydroxybenzoate, 0.059 g/l MgCl_2_.6H_2_O, 0.625 g/l KCl, 0.166 g/l CaCl_2_.2H_2_O, 0.326 g/l KH_2_PO_4_, and 0.804 g/l K_2_HPO_4_. The pH of the solution was adjusted to 6.75 using KOH [ 25 ].

The samples from the negative and positive control groups were immersed in their respective solutions for seven days at room temperature with daily renewal [ 26 ]. The remineralizing solution consisted of 0.4 M CaCl_2_, 0.04 M KH_2_PO_4_, 0.02 M NaF, and 8M NaCl, with a pH of 7.3 adjusted using 1M KOH [ 27 ].

Table 1 shows the characteristics of the materials used.

**Table 1:** The characteristics of the materials used

Manufactured by	Final volume	Description and composition	Material
Cariestop, Biodinâmica Química e Farmacêutica, Brazil	5ml	Silver Nitrate, Fluoridic acid, Amonia Hydroxide, Deionized Water	30% SDF
Pharmacy department laboratory of the Jundishapur University of Ahvaz, Iran	10 ml	Bioglass nanoparticle 45S5 (1gr), chitosan (2 ml)	Bioglass- chitosan solution
Primex, Iceland	10ml	Chitosan powder (25 mg), 0.1 M acetic acid (10 ml)	Chitosan solution (2.5 mg/ml)
laboratory of the Tehran University of medical sciences, Iran	10ml	Bioglass 45S5nanoparticles (0.6 g), distilled water (9.4 g)	Bioglass solution
Pharmacy department laboratory of the Jundishapur University of Ahvaz, Iran	400ml	CaCl_2_ (0.4 M), KH_2_PO_4_ (0.04 M), NaF (0.02 M), NaCl (8M), pH adjusted to 7.3 by 1M KOH	Remineralization solution
Pharmacy department laboratory of the Jundishapur University of Ahvaz, Iran	400ml	Acetic acid (0.2 M), KH_2_PO_4_(8.8 mM), CaCl_2_ (8.8 mM), pH adjusted to 4.4 by 4M KOH	Demineralization solution
Pharmacy department laboratory of the Jundishapur University of Ahvaz, Iran	1000ml	Sodium Carboxymethyl Cellulose (10 g/l), Methyl-phydroxybenzoate (2g/l), MgCl_2_.6H_2_O (0.059 g/l), KCl (0.625 g/l), CaCl_2_ .2H_2_O (0.166 g/l), KH_2_PO_4_ (0.326 g/l), K_2_HPO_4_ (0.804 g/l), pH adjusted to 6.75 by KOH	Artificial saliva

### Post-treatment Analysis

After the treatment, the samples were rinsed with distilled water and transported to the laboratory in a normal saline solution for secondary Vickers testing.

### Statistical Method

Continuous variables were reported as mean and standard deviation. The data normal distribution was assessed by the Shapiro-Wilk test. A two-way ANOVA test was performed for parametric data to examine the impact of two nominal predictor variables on a continuous outcome variable. Sidak's multiple comparisons test was conducted for pairwise comparisons. The significance level was set at *p*< 0.05. SPSS 22 (IBM SPSS Statistics, Armonk, New York, USA) and GraphPad Prism 8.4.2 were utilized for statistical analyses.

## Results

**Figure 1:**
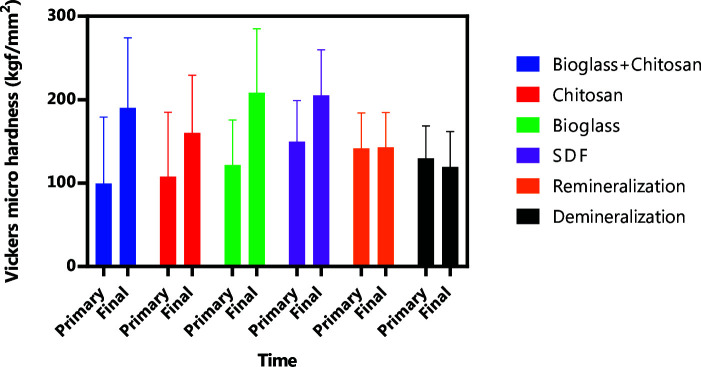
Vickers micro hardness values of the groups

**Figure 2:**
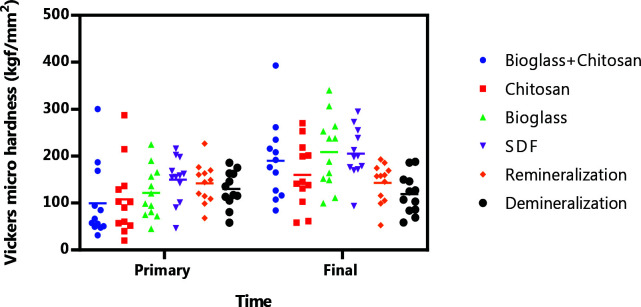
Distribution of the Vickers micro hardness values

The two-way ANOVA results demonstrated a statistically significant effect of the remineralizing compounds on both pre- and post-treatment microhardness (*p*< 0.0001). Tukey's post hoc test compared the primary and final VHN within each group. A significant difference was observed in the primary and final microhardness for the group 1(Bioglass-Chitosan group) (*p*< 0.0001), group 2 (Chitosan group) (*p*= 0.0006), group 3 (Bioglass group) (*p*< 0.0001), and group 4 (SDF group) (*p*= 0.0003). Nonetheless, no significant difference was detected in the primary and final VHN values for the group 5 (Remineralization group) (*p*> 0.9999) and the group 6 (Demineralization group) (*p*= 0.9554) (Table 2 and Figure 1). The microhardness difference between the studied groups was not statistically significant (*p*= 0.225), likely due to the dispersion of the samples. Figure 2 illustrates the distribution of these values.

**Table 2:** Vickers microhardness descriptive statistics of different groups

Demineralization	Remineralization	SDF	Bioglass	Chitosan	Bioglass+Chitosan	
129.8±38.6	141.7±42.2	149.7±49.2	121.6±53.9	107.7±77.1	99.4±79.6	Primary (mean±SD)
119.1±42.5	142.9±41.4	205.2±54.4	208.4±76.3	160.0±69.2	190.1±83.9	Final (mean±SD)
0.9554	>0.9999	0.0003*	<0.0001*	0.0006*	<0.0001*	*p* Value

* It is used to show a statistically significant difference

## Discussion

Dental caries are among the most common oral and dental diseases affecting adults and children [ 28 ]. A global study conducted in 2010 revealed that approximately 2.43 billion people worldwide are affected by dental caries [ 29 ]. In clinical observation, primary caries of tooth enamel presents as a white spot lesion. The white spot lesion is characterized by a partially intact surface layer formed through the re-deposition of dissolved phosphate and calcium ions, along with a porous lesion body. In the early stages, white spot lesion is considered reversible if remineralization processes can be stimulated [ 24 ]. 

Remineralization is a non-invasive and innovative approach for treating primary caries lesions. Its utilization bridges the gap between invasive and preventive dentistry [ 30 ]. The effectiveness of this treatment relies on the early detection of caries lesions and the accurate assessment of mineral loss to ensure appropriate interventions [ 31 ].

Cochrane *et al*. [ 32 ] provided a definition of remineralization as the process of crystal repair involving the deposition of minerals onto an enamel subsurface layer without the solid phase precipitation extending to the enamel surface layer.

Therefore, the deposition of minerals in the subsurface layer plays a crucial role in remineralizing primary caries lesions. While previous studies [ 6 - 7 ] have examined the effects of various remineralizing compounds, there has been no direct comparison of the effectiveness of chitosan, bioglass, chitosan-bioglass, and SDF.

In this investigation, the difference in Vickers microhardness between pre- and post-remineralization was 90.7 in the chitosan-bioglass group, 52.3 in the chitosan group, 86.8 in the bioglass group, 55.5 in the SDF group, 1.2 in the positive control group, and -10.7 in the negative control group (Table 2). With the exception of the positive and negative control groups, this difference was statistically significant in the remaining groups. Numerically, the largest change was observed in the Bioglass-Chitosan, Bioglass, SDF, and Chitosan groups, respectively. However, when comparing these groups statistically, no significant differences were found, which could be attributed to the dispersion of the data and the possibility of a small sample size.

In a study by Zhang *et al*. [ 5 ], the effect of various concentrations of bioglass 45S5 (2%, 4%, 6%, and 8%) on remineralization of enamel caries lesions in primary teeth was compared. The findings revealed that the 6% bioglass concentration yielded the most favorable mineral content and microhardness outcome. Therefore, a concentration of 6% bioglass was selected for the current experiment. Consistent with previous findings, the results of our study also demonstrated an increase in remineralization following treatment with 6% bioglass.

Punhagui *et al*. [ 33 ] compared SDF solutions of 30% and 38% using two different application times: 1 minute and 3 minutes. Their findings indicated that regardless of the SDF concentration, the application of SDF resulted in the remineralization of artificial caries lesions. Moreover, the group treated with a 30% SDF solution for 3 minutes exhibited less porosity than the other groups. Consistent with these findings, our study demonstrates that the application of SDF 30% for 3 minutes significantly enhances remineralization.

Ishikawa *et al*. [ 34 ] found that the use of bioglass-chitosan and bioglass resulted in significantly better improvements in the mechanical properties of enamel compared to chitosan alone. In a separate study, Zhang *et al*. [ 26 ] concluded that combining bioglass-chitosan outperformed the bioglass group in subsurface enamel remineralization. Similarly, Abbasi *et al*. [ 35 ] determined that bioglass paste 45S5 demonstrated effectiveness as a remineralizing agent for demineralized tooth enamel. These findings align with the results of our study.

In our study, the application of bioglass-chitosan, bioglass, and chitosan showed the highest impact on remineralization, which aligns with previous research findings. However, the comparison between the groups did not yield statistically significant results. This lack of significance may be attributed to variations in the testing methodology, sample size, and the method of material application.

In their study, Selma *et al*. [ 36 ] found that applying bioglass prior to enamel erosion can enhance enamel resistance against mineral loss in primary and permanent teeth. Based on these findings, bioglass 45S5 can serve as an effective preventive measure during periods of frequent consumption of acidic foods and beverages in children. It is worth noting that while Selma *et al*. [ 36 ] study utilized bioglass as a prophylactic measure before erosive lesions, our study focused on the use of bioglass after demineralization. Nonetheless, both studies demonstrated the effectiveness of bioglass in enhancing tooth resistance against acid attacks.

The results obtained from our study highlight the significant effectiveness of bioglass-chitosan and bioglass compounds in remineralizing primary caries lesions in primary teeth. These findings hold great promise for clinical applications and potential benefits in dental care.

Our study has several limitations, including challenges in ensuring uniform sample conditions, the omission of certain clinical factors like saliva enzymes, evaluation of the conditions after acid application, and difficulties in acquiring the necessary equipment for the study.

## Conclusion

All the materials utilized in the current study demonstrated a noteworthy remineralizing effect on primary caries lesions of primary teeth enamel. The chitosan-bioglass and bioglass groups exhibited the highest levels of remineralization. It is recommended that further studies be conducted under intraoral conditions to replicate the findings.
